# A Rare Encounter: Documenting a Rotator Cuff Tear Associated With Idiopathic Rice Bodies

**DOI:** 10.1155/cro/2370288

**Published:** 2026-06-05

**Authors:** Ekrem Özdemir, Oya Olcay Özdeş, Hüseyin Utku Özdeş

**Affiliations:** ^1^ Department of Orthopedics and Traumatology, Erzurum City Hospital, Erzurum, Türkiye; ^2^ Department of Anesthesia and Reanimation, Battalgazi State Hospital, Malatya, Türkiye; ^3^ Department of Orthopedics and Traumatology, İnönü University Medical School, Malatya, Türkiye

**Keywords:** rice body formation, rotator cuff injuries, shoulder joint

## Abstract

We share a case of a cuff tear caused by rice bodies, which typically occur in chronic inflammatory conditions and are often seen in autoimmune diseases. A 62‐year‐old patient presented with pain and restricted movement in the left shoulder. Magnetic resonance imaging revealed numerous rice body–like accumulations in the subacromial region, and a cuff tear was observed. The patient had no additional comorbidities in the medical history and no signs of infection on physical examination. Surgery was performed to repair the cuff rupture and excise the deposits. The histopathological examination of the excised material confirmed the presence of rice bodies. During the 20‐month follow‐up, the patient′s shoulder function improved and pain decreased. Although cuff tendinopathy is very common, cuff tears secondary to rice body synovitis and its mechanical effects are extremely rare. It should be noted that rice bodies developing in the context of chronic inflammation can present as a shoulder mass without systemic findings in the early stages. Although they may appear idiopathic, patients should be evaluated and counseled regarding possible autoimmune predisposition. In our case, infectious and autoimmune etiologies were carefully excluded through clinical, laboratory, and histopathological assessments.

## 1. Introduction

Rotator cuff tears are among the commonly seen orthopedic diseases and negatively affect the quality of life of patients. Pain while lying on the affected side at night, pain, and limitation in shoulder joint movements during daily activities are among the major symptoms. The etiology of cuff tears, which is well defined in the literature, has been associated with many pathologies such as impingement in the shoulder, acromial morphology, demographic characteristics, age‐related degeneration–microtrauma, oxidative stress, and vascular disorders [1, 2].

Rice body synovitis is a rarely seen disease. It has been shown to occur secondarily to infectious and rheumatological arthritis [3, 4]. In this article, we present rice bodies in the shoulder that exhibit clinical, radiological, and pathological features and do not develop secondarily to an underlying known disease. We share rice bodies that form without an inflammatory process and the resulting development of a rotator cuff tear. With this case, we aim to demonstrate that a rare cause like rice body synovitis can be found in the etiology of rotator cuff tears, in addition to the commonly encountered etiologies, and that rice bodies in the shoulder region may be idiopathic, serving as a model case for the diagnosis and treatment of patients.

## 2. Case Report

A 62‐year‐old female patient presented to the orthopedics clinic with complaints of severe night pain, swelling, and loss of function that had increased over the last 2 months, following an activity that began approximately 2 years ago in the left shoulder. The right‐handed patient had no history of trauma or previous surgeries. She had hypertension as a comorbidity. Blood tests, hemogram, and biochemical markers were within normal limits (WBC: 6.0 × 10^9^/L, Hb: 12.2 g/dL, platelets: 207 × 10^9^/L, erythrocyte sedimentation rate: 10 mm/h, CRP: 1 mg/L, RF: negative, and anti‐CCP: negative). Our patient had no history of tuberculosis. Clinically, she did not report cough, sputum production, or fever. The chest x‐ray showed no signs of active or previous tuberculosis. For preoperative evaluation, the patient was referred to the pulmonology department, and tuberculosis was not considered.

On physical examination, shoulder joint movements were painful during both active and passive ranges of motion in flexion, abduction, and internal rotation. Inspection revealed significant swelling in the left shoulder compared with the right, without local heat or redness. Range of motion was measured with a standard goniometer, and the recorded angles for flexion, abduction, and external rotation were 75°, 50°, and 25°, respectively, demonstrating a marked restriction in all planes of movement. Internal rotation was further limited, reaching only the level of the sacrum. The Jobe and Full Can tests were positive.

**Figure 1:**
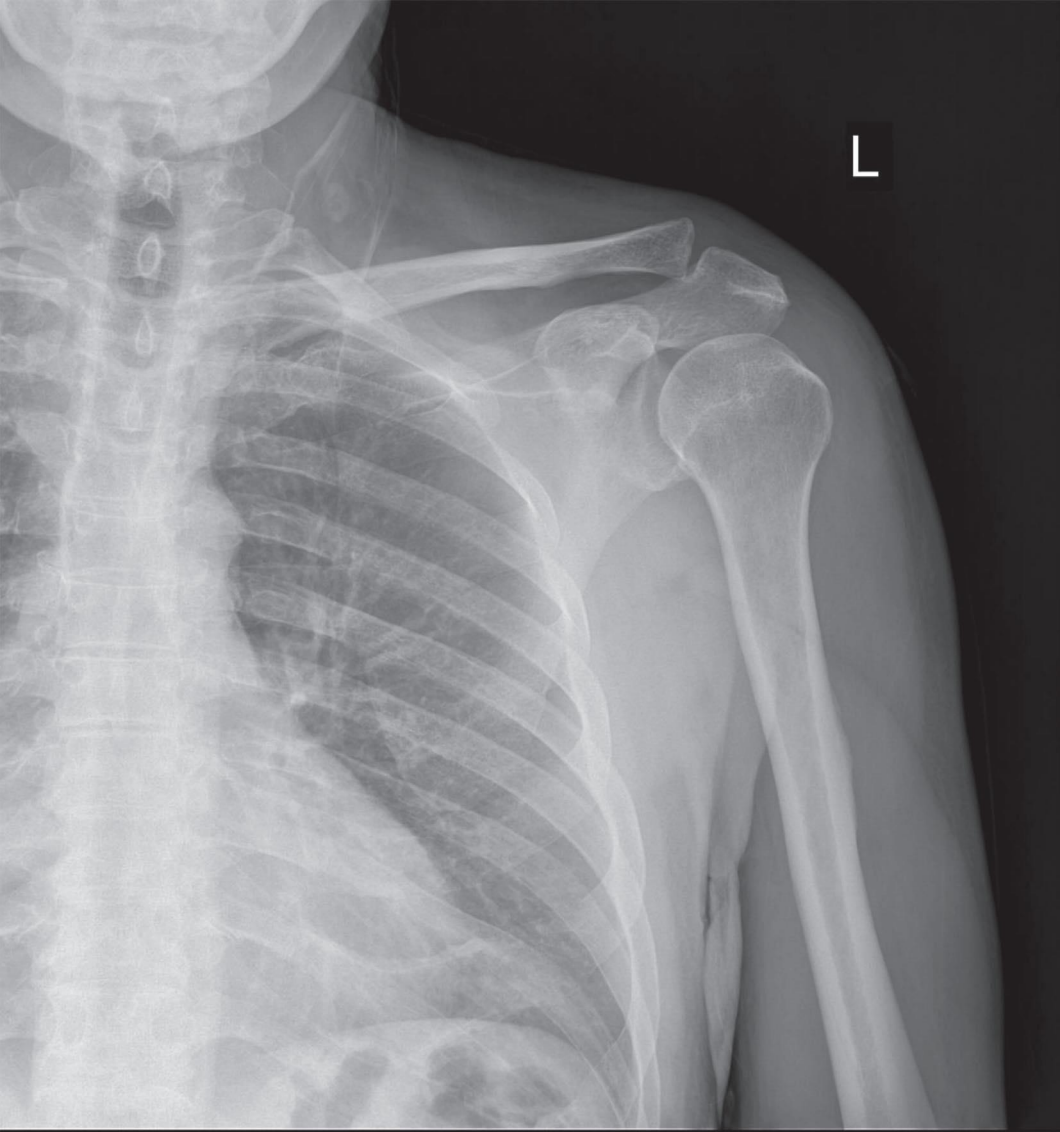
Anteroposterior x‐ray view of the shoulder demonstrating minimal subacromial space expansion accompanied by early‐stage degenerative (osteoarthritic) alterations of the glenohumeral joint.

**Figure 2:**
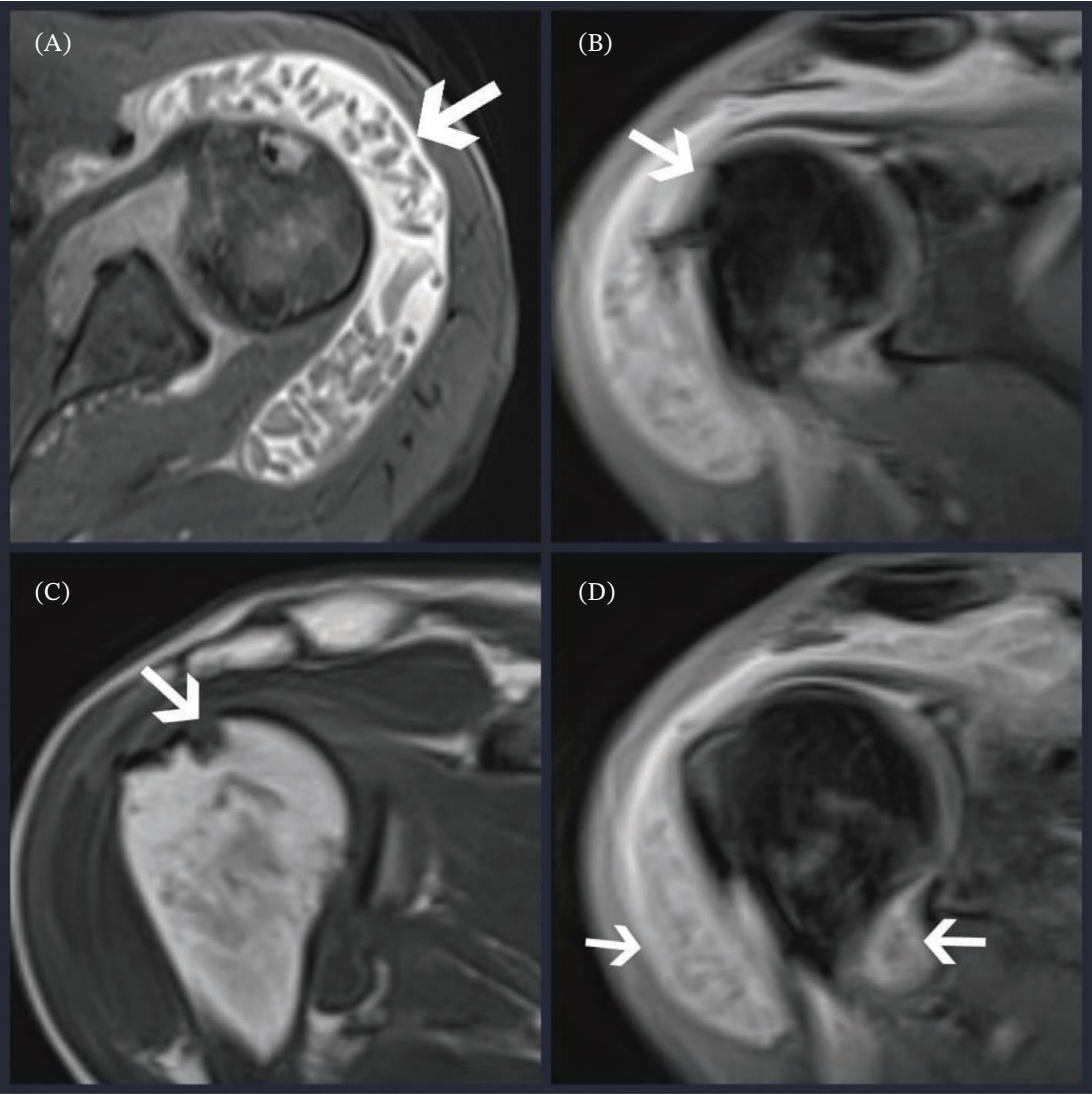
MRI findings. (A) Coronal T2‐weighted fat‐suppressed image showing multiple hypointense rice bodies within the subacromial–subdeltoid bursa (arrow). (B) Axial T2‐weighted fat‐suppressed image demonstrating a full‐thickness supraspinatus tendon tear (arrow). (C) Coronal T1‐weighted image revealing focal bone erosion at the humeral head (arrow). (D) Axial T2‐weighted fat‐suppressed image depicting diffuse synovial thickening and joint effusion (arrows).

In the shoulder x‐rays taken of the patient, there was widening in the subacromial space. The glenohumeral joint and acromial arch were evaluated as normal, and degenerative findings were not detected (Figure 1). Magnetic resonance imaging revealed multiple hypointense areas on T2‐weighted sequences, consistent with rice body–like structures diffusely distributed in the glenohumeral and subacromial spaces. A full‐thickness rupture of the supraspinatus tendon and localized bone erosions were also detected (Figure 2A–D). Based on the clinical findings, swelling, pain, restricted motion, and the MRI appearance, a preliminary diagnosis of rice body synovitis was made preoperatively. Surgical excision of the rice bodies and rotator cuff repair was, therefore, planned and discussed with the patient prior to surgery, after obtaining informed consent.

**Figure 3:**
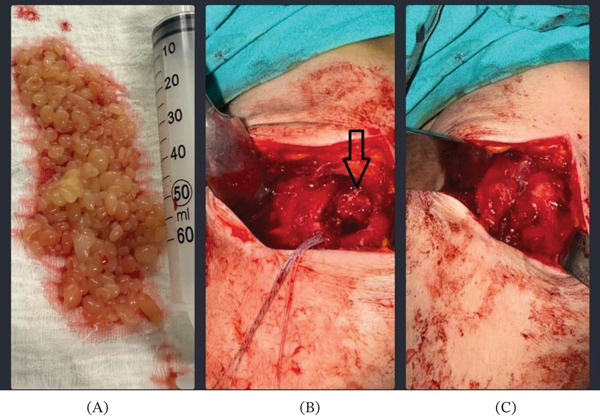
Macroscopic image of rice bodies. (A) The tear as seen before repair, (B) after the rice bodies were removed (black arrow), and (C) after repair.

An interscalene block was performed for postoperative pain control before surgery, and then the patient was taken to surgery in a supine position under general anesthesia. An approximately 5‐cm incision was made in the skin using the anterolateral transdeltoid approach, and the skin and subcutaneous tissues were dissected. The middle fibers of the deltoid were split. The subdeltoid bursa was resected, and the supraspinatus tendon was reached. A full‐thickness tear was confirmed intraoperatively. Numerous rice bodies, which showed extensive distribution toward the glenohumeral and subacromial areas, were present among the torn fibers of the supraspinatus. The rice bodies were completely excised along with the synovium, and subacromial decompression was performed. The rice bodies that were extracted were round or oval in shape, approximately less than 1 cm in size, and translucent white in color with a slightly adhesive texture. The excised material was sent to the pathology laboratory as a specimen for pathological diagnosis. An intraoperative full‐thickness tear of the rotator cuff was confirmed. The rotator cuff was repaired with one anchor and one push‐lock (Figure 3A–C). The deltoid was repaired. The skin and subcutaneous incisions were closed primarily. A dressing and Velpeau bandage were applied, and the operation was concluded. The patient was discharged 1 day after surgery. The sutures were removed in the second week. In the sixth postoperative week, the Velpeau bandage was discontinued, and the patient was referred to the physical therapy clinic for physical therapy protocols.

**Figure 4:**
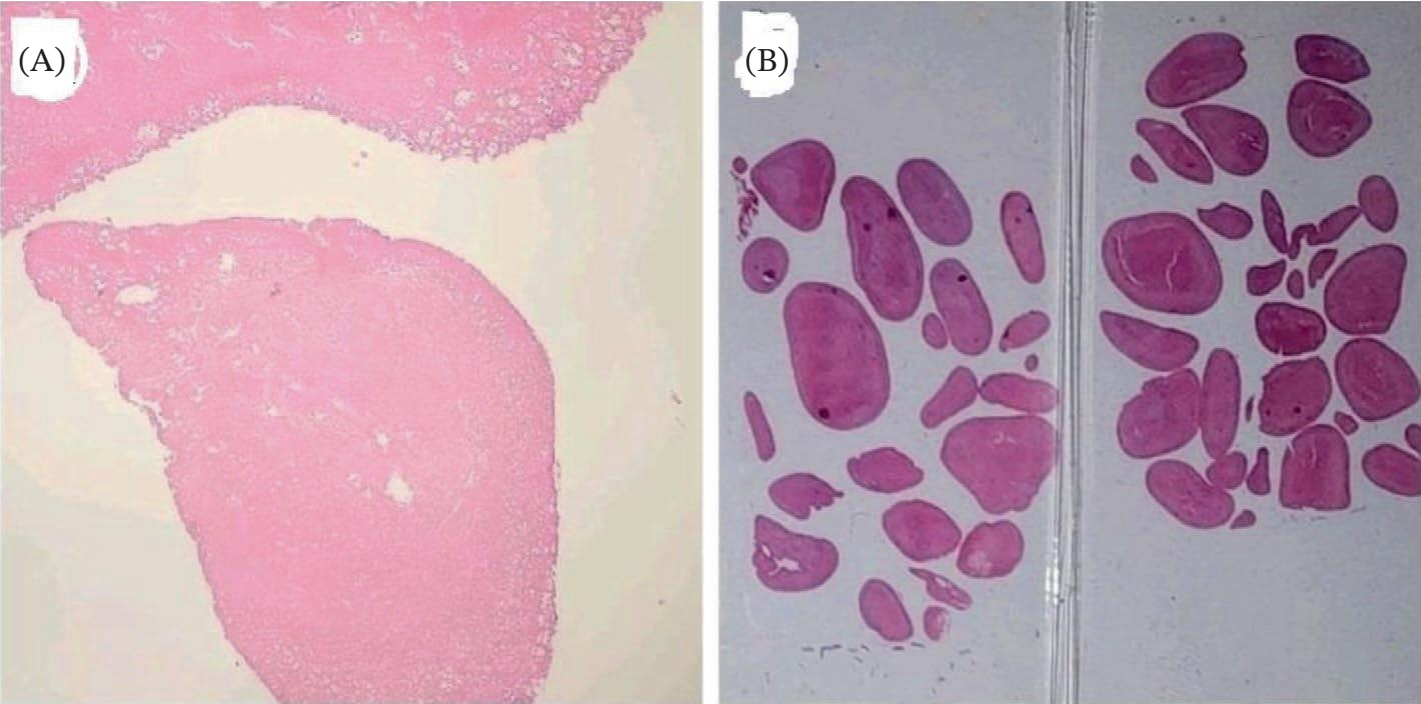
(A) Histopathological appearance of the rice bodies. The sections demonstrate that the structures are mainly composed of a dense fibrin material matrix with interspersed inflammatory cells, confirming the diagnosis of chronic rice body synovitis. (B) Rice bodies were fixed in formalin, embedded in paraffin, and sectioned into 4–5 *μ*m lamellar slices. Sections were stained with hematoxylin and eosin (H&E) for histopathological evaluation.

The excised biopsy materials were reported as rice bodies in synovitis (Figure 4A,B). Postoperative range of motion improved progressively at 6 weeks and 6 months and was maintained at 20 months, with pain decreasing and functional use of the arm increasing; internal rotation remained at the sacral level, but movement‐related pain resolved, and daily activities were performed without limitation. No recurrent lesions were detected in the follow‐up x‐rays and the MRI.

## 3. Discussion

Shoulder pain encountered in orthopedic clinics is increasingly framed under the umbrella term rotator cuff–related shoulder pain, which encompasses symptomatic rotator cuff pathology, including tendinopathy, bursitis, and rotator cuff tears. The etiology includes age‐related degeneration and micro‐ and macrotraumas and has also been associated with smoking, hypercholesterolemia, and genetic predisposition [5]. In the etiology of cuff tears, masses occupying space within the shoulder are quite rare compared to classical causes. In such cases, both the clinician and the patient focus on the masses, while the treatment of the cuff tear remains secondary. However, shoulder masses are generally benign in character, and the main findings are related to cuff tendinopathy. Cuff tears originating from pigmented villonodular synovitis, which have significant pain complaints, have been reported [6, 7]. Additionally, synovial cysts, acromioclavicular joint cysts, ganglion cysts, and cystic structures due to calcium pyrophosphate dihydrate accumulation can also present as clinical findings of cuff tears [8–10]. In our case, there were numerous rice bodies causing significant swelling in the shoulder. Although the patient′s complaint was due to a cuff tear, the palpable swelling and lesions revealed by imaging were concerning for the patient. Rice body particles, a rare lesion, can cause massive cuff tears by being located in the shoulder.

Rice bodies develop more commonly secondary to rheumatic and inflammatory arthritis. Rice body particles causing subacromial bursitis located in the shoulder have been surgically treated in a patient with rheumatoid arthritis [4, 11]. Rice bodies formed due to infectious sources have been shown to originate from chronic inflammation secondary to tuberculosis, but rice body formation due to candidiasis has also been described [3]. There is generally an immune reaction underlying rice bodies. In our case, detailed examination and the patient′s history did not reveal any autoimmune disease. Findings indicating inflammation, such as increased temperature and redness, were not detected. There is no septic shoulder joint. Complaints are entirely due to mechanical symptoms. The formation of this rare pathology has been interpreted as idiopathic during 20 months of follow‐up.

Rice bodies have a macroscopic appearance that is compact, smooth, and polished. In microscopic examination, there are findings of acute and/or chronic inflammation surrounded by fibrin and collagen, but they do not show microbial or pigment accumulation [12]. In suspected possible cuff tears identified by physical examination, MRI is the gold standard for detecting fatty atrophy, tendon retraction, and the tear itself. The tear, erosion in the bone, and rice body accumulation in soft tissues have been detected by MRI in our case. Rice bodies confirmed macroscopically by histopathological examination after surgery have been definitively identified. We think that the chronic inflammation seen in cuff tears that develop on the basis of degeneration without major trauma and the inflammation that causes rice body development may be related to each other, and we find it significant.

The treatment of rotator cuff tears is influenced by many factors, including the underlying cause, tear size and shape, mechanism of formation, and patient characteristics. Treatment decisions, whether conservative or surgical, are therefore individualized. In our case, because there was an underlying pathology that contributed to the rotator cuff tear and produced mechanical symptoms, surgical treatment was prioritized. Arthroscopic surgery is generally considered only in cases of rice body synovitis with a single or a small number of lesions [13]. In our patient, due to the extensive distribution of synovitis and its extension toward the distal joint, a miniopen approach was preferred. This technique provided wide exposure, facilitated excision, and offered practical advantages during the procedure.

This case report has several limitations. First, it is a retrospective study. Second, the 20‐month follow‐up period may be relatively short for fully evaluating the etiology and the development of rice bodies. Finally, postoperative shoulder range‐of‐motion measurements could not be retrieved from the outpatient clinic records. Although the patient′s clinical improvement, such as reduction of pain and swelling, was documented qualitatively, objective measurements and tests used to quantify shoulder motion were not available.

In conclusion, rotator cuff tears most commonly develop in older patients due to degenerative processes, and in some cases, additional mechanical factors, such as the rice bodies observed in our patient, may further contribute to the development of the tear by causing mechanical irritation. Rice bodies are typically associated with autoimmune rheumatic diseases and infectious conditions; however, as in our patient, they may occasionally appear idiopathically, without any underlying disease. Therefore, long‐term close follow‐up is recommended both to monitor for recurrence and to better understand the etiological factors involved.

This case report has been prepared in accordance with the CARE guidelines.

## Funding

No funding was received for this manuscript.

## Consent

Written informed consent was obtained from the patient.

## Conflicts of Interest

The authors declare no conflicts of interest.

## Supporting information


**Supporting Information** Additional supporting information can be found online in the Supporting Information section.

## Data Availability

The data that support the findings of this study are available from the corresponding author upon reasonable request.
